# Obesity and Multiple Sclerosis: A Mendelian Randomization Study

**DOI:** 10.1371/journal.pmed.1002053

**Published:** 2016-06-28

**Authors:** Lauren E. Mokry, Stephanie Ross, Nicholas J. Timpson, Stephen Sawcer, George Davey Smith, J. Brent Richards

**Affiliations:** 1 Department of Epidemiology, Biostatistics and Occupational Health, McGill University, Montreal, Quebec, Canada; 2 Centre for Clinical Epidemiology, Department of Epidemiology, Lady Davis Institute for Medical Research, Jewish General Hospital, McGill University, Montreal, Quebec, Canada; 3 MRC Integrative Epidemiology Unit, School of Social and Community Medicine, University of Bristol, Bristol, United Kingdom; 4 Department of Clinical Neurosciences, Cambridge Biomedical Campus, Cambridge, United Kingdom; 5 Department of Medicine, McGill University, Montreal, Quebec, Canada; 6 Department of Human Genetics, McGill University, Montreal, Quebec, Canada; 7 Department of Twin Research and Genetic Epidemiology, King's College London, London, United Kingdom; Imperial College London, UNITED KINGDOM

## Abstract

**Background:**

Observational studies have reported an association between obesity, as measured by elevated body mass index (BMI), in early adulthood and risk of multiple sclerosis (MS). However, bias potentially introduced by confounding and reverse causation may have influenced these findings. Therefore, we elected to perform Mendelian randomization (MR) analyses to evaluate whether genetically increased BMI is associated with an increased risk of MS.

**Methods and Findings:**

Employing a two-sample MR approach, we used summary statistics from the Genetic Investigation of Anthropometric Traits (GIANT) consortium and the International MS Genetics Consortium (IMSGC), the largest genome-wide association studies for BMI and MS, respectively (GIANT: *n* = 322,105; IMSGC: *n* = 14,498 cases and 24,091 controls). Seventy single nucleotide polymorphisms (SNPs) were genome-wide significant (*p* < 5 x 10^−8^) for BMI in GIANT (*n* = 322,105) and were investigated for their association with MS risk in the IMSGC. The effect of each SNP on MS was weighted by its effect on BMI, and estimates were pooled to provide a summary measure for the effect of increased BMI upon risk of MS. Our results suggest that increased BMI influences MS susceptibility, where a 1 standard deviation increase in genetically determined BMI (kg/m^2^) increased odds of MS by 41% (odds ratio [OR]: 1.41, 95% CI 1.20–1.66, *p* = 2.7 x 10^−5^, I^2^ = 0%, 95% CI 0–29). Sensitivity analyses, including MR-Egger regression, and the weighted median approach provided no evidence of pleiotropic effects. The main study limitations are that, while these sensitivity analyses reduce the possibility that pleiotropy influenced our results, residual pleiotropy is difficult to exclude entirely.

**Conclusion:**

Genetically elevated BMI is associated with risk of MS, providing evidence for a causal role for obesity in MS etiology. While obesity has been associated with many late-life outcomes, these findings suggest an important consequence of childhood and/or early adulthood obesity.

## Abstract 2

Using a Mendelian randomization approach, Brent Richards and colleagues examine the possibility that genetically raised body mass index could affect risk of multiple sclerosis.

## Author Summary

**Why Was This Study Done?:**

- Multiple sclerosis (MS) is a debilitating disease that carries a large social and economic burden.
- The risk factors that cause MS remain poorly understood.
- Previous observational epidemiological studies have reported an association between elevated body mass index (BMI) in early adulthood and risk of MS; however, lifestyle factors that influence BMI may bias the relationship between BMI and MS.

**What Did the Researchers Do and Find?:**

- The researchers tested whether inherited genetic variation that influences BMI is associated with MS. Such analyses provide an estimate of the relationship between BMI and MS that is not influenced by confounding factors, with the exception of confounding by ancestry; since assignment to genotype at conception is a random process, it breaks associations with other potential confounding factors.
- Using data from the largest genome-wide association study consortia for MS and BMI, the researchers provided evidence supporting elevated BMI as a causal risk factor for MS.
- A genetically determined change in the BMI category from overweight to obese was associated with a substantially increased risk of MS in this study.

**What Do These Findings Mean?:**

- Elevated BMI could be an important, and potentially modifiable, risk factor for MS.
- This provides further rationale to address rising obesity rates and to investigate whether interventions that promote a healthy lifestyle may help to mitigate MS risk.

## Introduction

Multiple sclerosis (MS) is a debilitating autoimmune disease of the central nervous system that results in chronic disability for the majority of those affected [1]. The disease has an important impact on the health economy of many countries [2], since current treatment regimens are costly and have adverse side-effect profiles and/or limited efficacy [3]. MS etiology is poorly understood and, consequently, additional research is needed to identify potentially causal risk factors that could help guide prevention efforts.

Several observational studies have suggested that an elevated body mass index (BMI) in early adulthood is associated with an increased risk of developing MS [4–6]. In the Nurse’s Health study, a prospective cohort study of 238,371 women, BMI ≥ 30 kg/m^2^ at 18 y of age was associated with more than a 2-fold increased risk of MS (relative risk: 2.25, 95% CI 1.50–3.37) [4]. In addition, elevated BMI has been shown to affect the immune system by promoting a proinflammatory state [7–10], and it has been proposed that adipose-derived hormones, such as leptin [11] and adiponectin [12], might mediate this, providing a possible mechanistic link between obesity and risk of MS.

**Fig 1:**
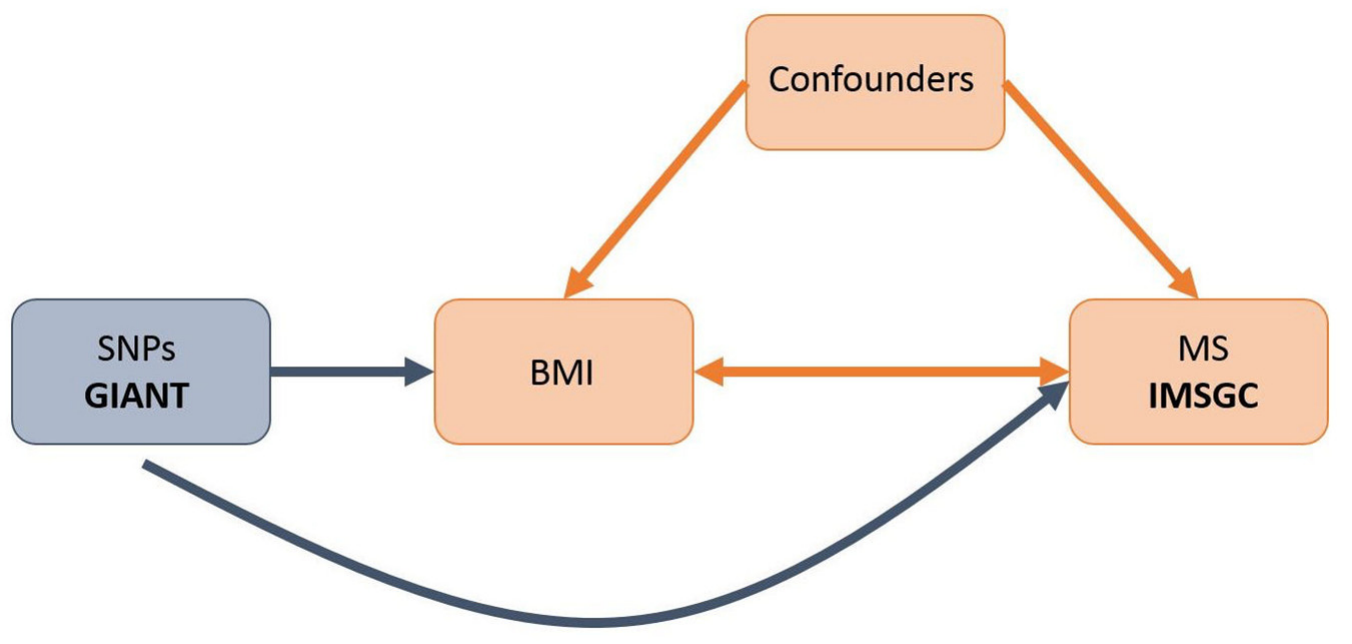
Schematic representation of an MR analysis. This diagram shows that SNPs associated with BMI were selected from the GIANT consortium. Corresponding effect estimates for these SNPs upon risk of MS were obtained from the IMSGC. Because of the randomization of alleles at meiosis, SNPs are not associated with confounding variables that may bias estimates obtained from observational studies.

However, it remains uncertain whether this relationship is causal since it is difficult to fully protect observational studies from bias due to reverse causation or confounding. Mendelian randomization (MR) [13] offers a way to investigate potentially causal relationships by using genetic associations to explore the effect of modifiable exposures on outcomes. Since alleles are both independently segregated and randomly assigned at meiosis, bias inherent to observational study designs, such as confounding, is likely to be greatly minimized in MR studies (Fig 1).

While increased BMI in childhood has been associated with numerous poor health outcomes [14,15], efforts to control obesity in children has been hindered by a lack of motivation in youth, due in part to the perception that most obesity-related diseases have a late age of onset [16]. However, if obesity were causally associated with MS (median age of onset 29 y) [17], this would provide a more immediate and severe consequence of early-life obesity, increasing motivation for its control.

In order to test whether genetically increased BMI is causally associated with risk of MS, we undertook an MR analysis using summary statistics from the Genetic Investigation of Anthropometric Traits (GIANT) consortium (up to *n* = 339,224) [18] and the International Multiple Sclerosis Genetics Consortium (IMSGC, up to *n* = 14,498 cases and 24,091 controls) [19,20], the largest genome-wide association studies (GWAS) to date for BMI and MS, respectively.

## Methods

### Data Sources and Single Nucleotide Polymorphism (SNP) Selection

Effect estimates for SNPs associated with BMI were obtained from the GIANT consortium, which is a meta-analysis of 125 GWASs in more than 339,224 individuals [18]. For the purpose of this MR, we limited our selection of SNPs to those that achieved genome-wide significance (*p* < 5 x 10^−8^) in the European sex-combined analysis (up to *n* = 322,105). Effect estimates of these BMI-associated SNPs on the risk of MS were assessed using the summary statistics from the IMSGC Immunochip study, which included 14,498 cases and 24,091 controls of European ancestry [19]. When effect estimates were not available in the Immunochip study, since the genotyping platform used was not genome-wide, effect estimates were obtained from the IMSGC/Wellcome Trust Case Control Consortium 2 (IMSGC/WTCCC2) GWAS, which included 9,772 cases and 17,376 controls [20].

When a BMI-associated SNP was not present in either study, we selected proxy SNPs that were highly correlated with the variant of interest (*r* ^2^ > 0.8). We identified potential proxies and measured linkage disequilibrium (LD) in samples of European descent from the UK10K consortium (*n* = 3,781) [21]. If no proxy was found using this method, we verified our search using SNAP [22,23]. As we did with our index SNPs, we selected summary statistics for our proxies first from the Immunochip study and then from the IMSGC/WTCCC2 study if they were not available in the Immunochip study.

Cohorts participating in the IMSGC and GIANT GWAS consortia received ethics approval from local institutional review boards and informed consent from all participants. Summary statistics from these consortia can be downloaded at the following links: IMSGC, https://www.immunobase.org; GIANT, https://www.broadinstitute.org/collaboration/giant/index.php/GIANT_consortium.

### SNP Validation

#### LD assessment

One condition of MR is that the exposure-related SNPs (the instrumental variables) must not be in LD with each other, since this might result in confounding if a SNP is highly correlated with another SNP used as an instrumental variable [24]. To ensure this, we measured LD between all of our selected SNPs in European samples from UK10K (*n* = 3,781) [21] using PLINK software version 1.90 [25]. SNPs were removed from our analysis if their measured LD had an *r* ^2^ greater than 0.05. We retained the SNP most strongly associated with BMI, by *p*-value, when two, or more, SNPs were in LD.

#### Pleiotropy assessment

An important assumption of MR is that each SNP must only influence risk of the outcome through the exposure under investigation, as the inclusion of SNPs that contribute through a pleiotropic pathway could bias estimates. This can be difficult to test when examining an anthropometric trait such as BMI, which is influenced by many different physiological mechanisms. Consequently, BMI-associated SNPs are likely to lie in genes of different biological effects that may, or may not, influence MS risk independently of BMI. To assess for the presence of pleiotropy, we used MR-Egger regression [26]. In brief, the approach is based on Egger regression, which has been used to examine publication bias in the meta-analysis literature [27]. Using the MR-Egger method, the SNP’s effect upon the exposure variable is plotted against its effect upon the outcome, and an intercept distinct from the origin provides evidence for pleiotropic effects. Funnel plots can also be used for visual inspection of symmetry. Additionally, the slope of the MR-Egger regression can provide pleiotropy-corrected causal estimates; however, this estimate of slope is underpowered unless the SNPs combine to explain a large proportion of the variance in the exposure with varying effect sizes [26]. An important condition of this approach is that a SNP’s association with the exposure variable must be independent of its direct effects upon the outcome (previously described as the InSIDE assumption) [26], which may not always be satisfied in cases in which all pleiotropic effects can be attributed to a single confounder. Nonetheless, the MR-Egger method can provide unbiased estimates even if all the chosen SNPs are invalid [26].

Additionally, the weighted median approach was used to examine pleiotropy [28]. Using this method, MR estimates are ordered and weighted by the inverse of their variance. The median MR estimate should remain unbiased as long as greater than 50% of the total weight comes from SNPs without pleiotropic effects. The weighted median approach offers some important advantages over MR-Egger because it improves precision and is more robust to violations of the InSIDE assumption. Therefore, we employed both methods as sensitivity analyses to assess whether pleiotropy had influenced our results.

#### Population stratification assessment

Population stratification is another potential source of bias for MR analyses. Because of differences in allele frequencies, a SNP can be associated with ancestry, which itself can be associated with disease risk. To limit this, we selected SNPs and summary statistics from analyses that included only individuals of European descent for both BMI and MS. However, residual population stratification may exist among European subgroups [29]. Therefore, to understand how this may affect our results, we searched the literature to explore whether BMI and MS covary geographically within Europe.

### MR Estimates

We have previously applied the principles of MR to investigate the role of vitamin D in the etiology of MS [30]. Here, we employed the same methods to examine BMI as a risk factor for MS [30–32]. In brief, we selected SNPs that were genome-wide significant for BMI in the GIANT consortium and then obtained the effect estimates of these SNPs on BMI. Corresponding genetic effect estimates for these SNPs on the risk of MS were selected from the IMSGC. We then applied a two-sample MR approach by weighting the effect estimate of each SNP on MS by its effect on BMI. These estimates were then pooled using a fixed meta-analytic model [33,34] to produce a summary measure of the effect of genetically elevated BMI upon MS risk. This two-sample approach has equivalent statistical power to one-sample approaches [31] and is favorable in this setting since large GWAS consortia exist for both BMI and MS and are thus better powered than an MR study in a single cohort with a smaller sample size.

### Bidirectional MR Analysis

Next, we sought to explore whether MS influences BMI. Therefore, we elected to perform a bidirectional MR analysis to determine the effect of genetically increased risk of MS on BMI. To do so, we selected SNPs that were genome-wide significant (*p* < 5 x 10^−8^) for MS in either the Immunochip study, the IMSGC/WTCCC2 study, or other previously reported MS GWASs [19,20,35]. With MS-associated SNPs now as the exposure, we then obtained corresponding effect estimates from GIANT as the outcome. We next applied the same methods as above.

### Sensitivity Analyses

To ensure that the inclusion of proxy SNPs did not introduce random error into our results, we conducted sensitivity analyses where groups of SNPs were excluded. First, we removed all proxies and retained only SNPs that had been directly genotyped in the Immunochip or IMSGC/WTCCC2 studies. Next, we excluded only proxies with allele frequencies between 0.4 and 0.6, as it can be difficult to correctly match alleles for SNPs falling within this range since different GWAS studies report slight fluctuations in allele frequencies. Since BMI is calculated using both height and weight measurements, we were concerned that our results might be partially confounded by height. Therefore, we next removed SNPs from our analysis that were found to be in LD (*r* ^*2*^ > 0.05) with any loci genome-wide significant for height as reported in GIANT’s 2014 GWAS [36]. Lastly, we performed an analysis in which genetic effect sizes on MS were selected exclusively from the IMSGC/WTCCC2 study.

## Results

### SNP Selection

Overall, we identified 77 LD-independent SNPs that achieved genome-wide significance for BMI in the GIANT consortium. However, not all of these SNPs were genotyped in the MS datasets. Fourteen of these SNPs were included in the IMSGC Immunochip study, and a further 20 in the IMSGC/WTCCC2 GWAS. Of the remaining SNPs, we next identified 36 proxies with an *r* ^2^ > 0.8: 12 from the IMSGC Immunochip study and 24 from the IMSGC/WTCCC2 GWAS. We could not identify proxies for seven of the BMI SNPs. A flow diagram of this SNP selection process is shown in S1 Fig. Therefore, in total we used 70 SNPs for our MR analysis, as shown in Table 1. The mean *r* ^2^ for proxies was 0.94.

**Table 1:** Characteristics of SNPs used in MR analysis.

					BMI Results		MS Results						
BMI-Associated SNP	Nearest Gene(s)	Chr	BMI Increasing Allele	Allele Frequency ^a^	Effect on BMI^a^	*p*-value^a^	OR^b^	Lower 95% CI^b^	Upper 95% CI^b^	*p*-value^b^	Study^b^	Proxy SNP	*r* ^2^ ^c^
rs1558902	*FTO*	16	A	0.42	0.082	7.5 x 10^−153^	1.00	0.97	1.04	0.87	Immunochip	NA	1.00
rs6567160	*MC4R*	18	C	0.24	0.056	3.9 x 10^−53^	1.00	0.96	1.04	0.97	Immunochip	rs17782313	0.99
rs13021737	*TMEM18*	2	G	0.83	0.060	1.1 x 10^−50^	1.05	1.01	1.10	0.016	Immunochip	rs6548238	0.99
rs10938397	*GNPDA2*	4	G	0.43	0.040	3.2 x 10^−38^	1.02	0.99	1.05	0.28	Immunochip	NA	1.00
rs543874	*SEC16B*	1	G	0.19	0.048	2.6 x 10^−35^	1.00	0.96	1.05	0.86	WTCCC2	rs633715	0.95
rs2207139	*TFAP2B*	6	G	0.18	0.045	4.1 x 10^−29^	1.04	0.99	1.08	0.11	Immunochip	rs987237	0.92
rs11030104	*BDNF*	11	A	0.79	0.041	5.6 x 10^−28^	1.03	0.99	1.08	0.16	WTCCC2	NA	1.00
rs3101336	*NEGR1*	1	C	0.61	0.033	2.7 x 10^−26^	0.99	0.96	1.03	0.73	Immunochip	rs2815752	1.00
rs7138803	*BCDIN3D*	12	A	0.38	0.032	8.2 x 10^−24^	1.02	0.98	1.06	0.31	WTCCC2	NA	1.00
rs10182181	*ADCY3*	2	G	0.46	0.031	8.8 x 10^−24^	1.06	1.02	1.09	8.8 x 10^−4^	Immunochip	NA	1.00
rs3888190	*ATP2A1*	16	A	0.40	0.031	3.1 x 10^−23^	1.01	0.97	1.04	0.69	Immunochip	NA	1.00
rs1516725	*ETV5*	3	G	0.87	0.045	1.9 x 10^−22^	1.02	0.97	1.08	0.41	WTCCC2	rs6809651	0.99
rs12446632	*GPRC5B*	16	G	0.87	0.040	1.5 x 10^−18^	1.06	1.00	1.12	0.038	WTCCC2	NA	1.00
rs2287019	*QPCTL*	19	C	0.80	0.036	4.6 x 10^−18^	1.05	1.00	1.09	0.037	WTCCC2	NA	1.00
rs16951275	*MAP2K5*	15	T	0.78	0.031	1.9 x 10^−17^	0.99	0.95	1.03	0.53	Immunochip	rs28670272	0.98
rs3817334	*MTCH2*	11	T	0.41	0.026	5.2 x 10^−17^	0.97	0.94	1.01	0.14	WTCCC2	rs7124681	0.99
rs2112347	*POC5*	5	T	0.63	0.026	6.2 x 10^−17^	1.02	0.98	1.05	0.38	Immunochip	rs34358	0.85
rs12566985	*FPGT-TNNI3K*	1	G	0.45	0.024	3.3 x 10^−15^	0.99	0.96	1.03	0.76	WTCCC2	rs6604872	1.00
rs3810291	*ZC3H4*	19	A	0.67	0.028	4.8 x 10^−15^	0.97	0.94	1.01	0.11	Immunochip	rs10408163	1.00
rs7141420	*NRXN3*	14	T	0.53	0.024	1.2 x 10^−14^	1.02	0.99	1.06	0.21	WTCCC2	NA	1.00
rs13078960	*CADM2*	3	G	0.20	0.030	1.7 x 10^−14^	0.99	0.95	1.04	0.69	WTCCC2	rs7622475	0.99
rs10968576	*LINGO2*	9	G	0.32	0.025	6.6 x 10^−14^	1.01	0.97	1.05	0.54	WTCCC2	NA	1.00
rs12429545	*OLFM4*	13	A	0.13	0.033	1.1 x 10^−12^	1.01	0.96	1.06	0.79	WTCCC2	NA	1.00
rs12286929	*CADM1*	11	G	0.52	0.022	1.3 x 10^−12^	0.99	0.95	1.03	0.57	WTCCC2	rs12421648	0.84
rs11165643	*PTBP2*	1	T	0.58	0.022	2.1 x 10^−12^	1.01	0.97	1.05	0.69	WTCCC2	NA	1.00
rs7903146	*TCF7L2*	10	C	0.71	0.023	1.1 x 10^−11^	0.98	0.95	1.02	0.35	WTCCC2	NA	1.00
rs10132280	*STXBP6*	14	C	0.68	0.023	1.1 x 10^−11^	1.00	0.96	1.03	0.84	Immunochip	rs12432376	0.84
rs17405819	*HNF4G*	8	T	0.70	0.022	2.1 x 10^−11^	0.99	0.95	1.03	0.71	WTCCC2	rs2977345	0.97
rs1016287	*LINC01122*	2	T	0.29	0.023	2.3 x 10^−11^	1.03	0.99	1.07	0.11	WTCCC2	rs759250	1.00
rs4256980	*TRIM66*	11	G	0.65	0.021	2.9 x 10^−11^	0.99	0.95	1.03	0.69	WTCCC2	rs2316901	0.99
rs17094222	*HIF1AN*	10	C	0.21	0.025	5.9 x 10^−11^	1.01	0.97	1.06	0.58	WTCCC2	rs17113301	0.90
rs12401738	*FUBP1*	1	A	0.35	0.021	1.2 x 10^−10^	1.01	0.97	1.04	0.63	Immunochip	rs4130548	1.00
rs7599312	*ERBB4*	2	G	0.72	0.022	1.2 x 10^−10^	1.02	0.98	1.07	0.24	WTCCC2	NA	1.00
rs2365389	*FHIT*	3	C	0.58	0.020	1.6 x 10^−10^	0.98	0.94	1.02	0.29	WTCCC2	rs7629340	0.98
rs205262	*C6orf106*	6	G	0.27	0.022	1.8 x 10^−10^	1.01	0.97	1.05	0.62	Immunochip	NA	1.00
rs2820292	*NAV1*	1	C	0.56	0.020	1.8 x 10^−10^	1.03	0.99	1.07	0.11	WTCCC2	rs1032524	0.93
rs12885454	*PRKD1*	14	C	0.64	0.021	1.9 x 10^−10^	1.01	0.98	1.05	0.42	WTCCC2	rs1307813	1.00
rs12016871	*MTIF3*	13	T	0.20	0.030	2.3 x 10^−10^	1.01	0.97	1.06	0.58	WTCCC2	rs1885989	0.82
rs16851483	*RASA2*	3	T	0.07	0.048	3.6 x 10^−10^	1.02	0.95	1.09	0.63	WTCCC2	rs2640017	0.99
rs1167827	*HIP1*	7	G	0.55	0.020	6.3 x 10^−10^	1.00	0.97	1.04	0.93	Immunochip	NA	1.00
rs758747	*NLRC3*	16	T	0.27	0.023	7.5 x 10^−10^	1.03	0.99	1.07	0.16	WTCCC2	NA	1.00
rs1928295	*TLR4*	9	T	0.55	0.019	7.9 x 10^−10^	1.02	0.99	1.06	0.25	WTCCC2	NA	1.00
rs9925964	*KAT8*	16	A	0.62	0.019	8.1 x 10^−10^	1.02	0.98	1.05	0.36	Immunochip	NA	1.00
rs11126666	*KCNK3*	2	A	0.28	0.021	1.3 x 10^−9^	1.02	0.99	1.06	0.25	Immunochip	NA	1.00
rs2650492	*SBK1*	16	A	0.30	0.021	1.9 x 10^−9^	1.01	0.98	1.05	0.58	Immunochip	NA	1.00
rs6804842	*RARB*	3	G	0.58	0.019	2.5 x 10^−9^	1.03	0.99	1.07	0.15	WTCCC2	NA	1.00
rs12940622	*RPTOR*	17	G	0.58	0.018	2.5x10^-9^	1.01	0.97	1.05	0.58	WTCCC2	NA	1.00
rs4740619	*C9orf93*	9	T	0.54	0.018	4.6x10^-9^	1.00	0.96	1.04	0.86	WTCCC2	NA	1.00
rs13191362	*PARK2*	6	A	0.88	0.028	7.3 x 10^−9^	1.01	0.95	1.07	0.83	WTCCC2	rs13202339	0.98
rs3736485	*DMXL2*	15	A	0.45	0.018	7.4 x 10^−9^	0.97	0.94	1.00	0.11	Immunochip	rs4775961	0.88
rs17001654	*SCARB2*	4	G	0.15	0.031	7.7 x 10^−9^	1.04	1.00	1.09	0.089	Immunochip	rs17001561	0.95
rs11191560	*NT5C2*	10	C	0.09	0.031	8.5 x 10^−9^	0.98	0.93	1.04	0.58	Immunochip	NA	1.00
rs1528435	*UBE2E3*	2	T	0.63	0.018	1.2 x 10^−8^	1.00	0.97	1.04	0.83	WTCCC2	rs6727573	0.95
rs2075650	*TOMM40*	19	A	0.85	0.026	1.3 x 10^−8^	1.03	0.98	1.08	0.21	Immunochip	NA	1.00
rs1000940	*RABEP1*	17	G	0.32	0.019	1.3 x 10^−8^	1.01	0.97	1.05	0.64	WTCCC2	NA	1.00
rs11583200	*ELAVL4*	1	C	0.40	0.018	1.5 x 10^−8^	1.01	0.98	1.05	0.48	WTCCC2	NA	1.00
rs9400239	*FOXO3*	6	C	0.69	0.019	1.6 x 10^−8^	0.97	0.93	1.01	0.15	WTCCC2	rs2153960	0.93
rs10733682	*LMX1B*	9	A	0.48	0.017	1.8 x 10^−8^	1.00	0.97	1.04	0.96	WTCCC2	NA	1.00
rs11688816	*EHBP1*	2	G	0.53	0.017	1.9 x 10^−8^	1.02	0.99	1.06	0.21	WTCCC2	rs360791	0.82
rs11057405	*CLIP1*	12	G	0.90	0.031	2.0 x 10^−8^	1.05	0.99	1.11	0.074	Immunochip	NA	1.00
rs2121279	*LRP1B*	2	T	0.12	0.025	2.3 x 10^−8^	1.02	0.96	1.08	0.53	WTCCC2	rs6714473	0.80
rs29941	*KCTD15*	19	G	0.67	0.018	2.4 x 10^−8^	1.00	0.97	1.04	0.86	Immunochip	NA	1.00
rs3849570	*GBE1*	3	A	0.36	0.019	2.6 x 10^−8^	1.00	0.96	1.03	0.86	Immunochip	rs3860595	0.88
rs6477694	*EPB41L4B*	9	C	0.37	0.017	2.7 x 10^−8^	1.02	0.98	1.05	0.33	Immunochip	NA	1.00
rs2176598	*HSD17B12*	11	T	0.25	0.020	3.0 x 10^−8^	1.03	0.99	1.07	0.16	WTCCC2	rs7110437	0.81
rs7899106	*GRID1*	10	G	0.05	0.040	3.0 x 10^−8^	1.06	0.98	1.15	0.15	WTCCC2	rs11201714	1.00
rs17724992	*PGPEP1*	19	A	0.75	0.019	3.4 x 10^−8^	1.02	0.98	1.06	0.34	WTCCC2	NA	1.00
rs7243357	*GRP*	18	T	0.81	0.022	3.9 x 10^−8^	0.99	0.95	1.04	0.70	WTCCC2	rs9961404	0.90
rs1808579	*C18orf8*	18	C	0.53	0.017	4.2 x 10^−8^	1.00	0.96	1.04	0.91	WTCCC2	NA	1.00
rs2033732	*RALYL*	8	C	0.75	0.019	4.9 x 10^−8^	1.00	0.96	1.04	0.83	WTCCC2	rs733594	0.91

^a^ The allele frequency, effect size (β-coefficient, measured as a standard deviation [SD] change per additional BMI increasing allele), and *p*-value for each SNP were obtained from the GIANT consortium European sex-combined analysis [18].

^b^ Immunochip refers to the IMSGC Immunochip study, and WTCCC2 refers to the IMSGC/WTCCC2 study. The OR, 95% CI, and *p*-value for a BMI SNP’s association with MS were selected from either of these studies.

^c^ r^2^ were estimated using UK10K European samples [21].

### SNP Validation

**Fig 2:**
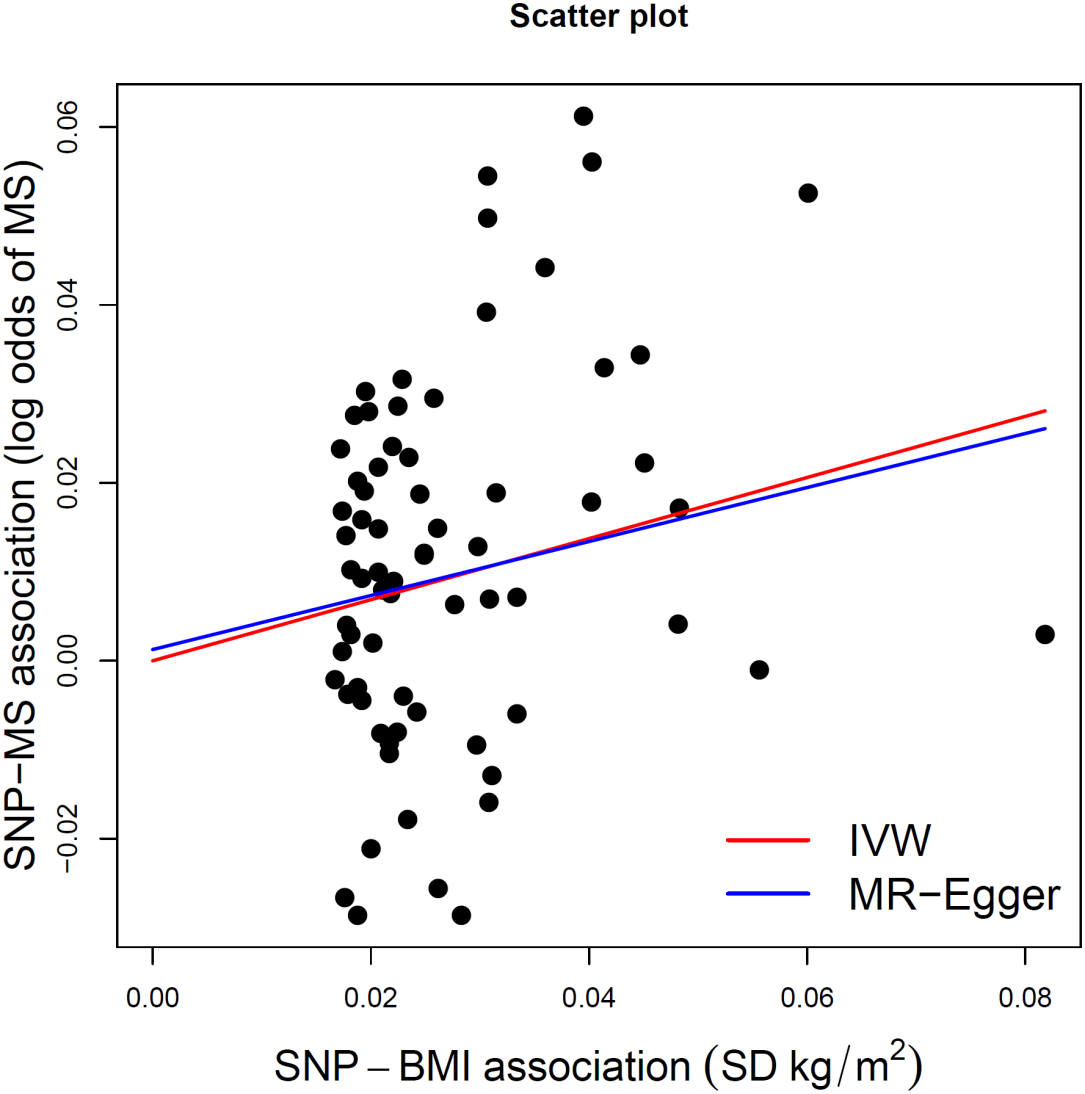
MR-Egger regression scatterplot for BMI on MS analysis. The red line shows the results of standard MR analysis (inverse-variance weighting [IVW]), and the blue line shows the pleiotropy-adjusted MR-Egger regression line. The estimated slope of the MR-Egger regression, expressed as an OR, was 1.35 (95% CI 0.91–2.02). The estimated MR-Egger intercept term was 0.0013 (95% CI −0.010–0.013)

**Fig 3:**
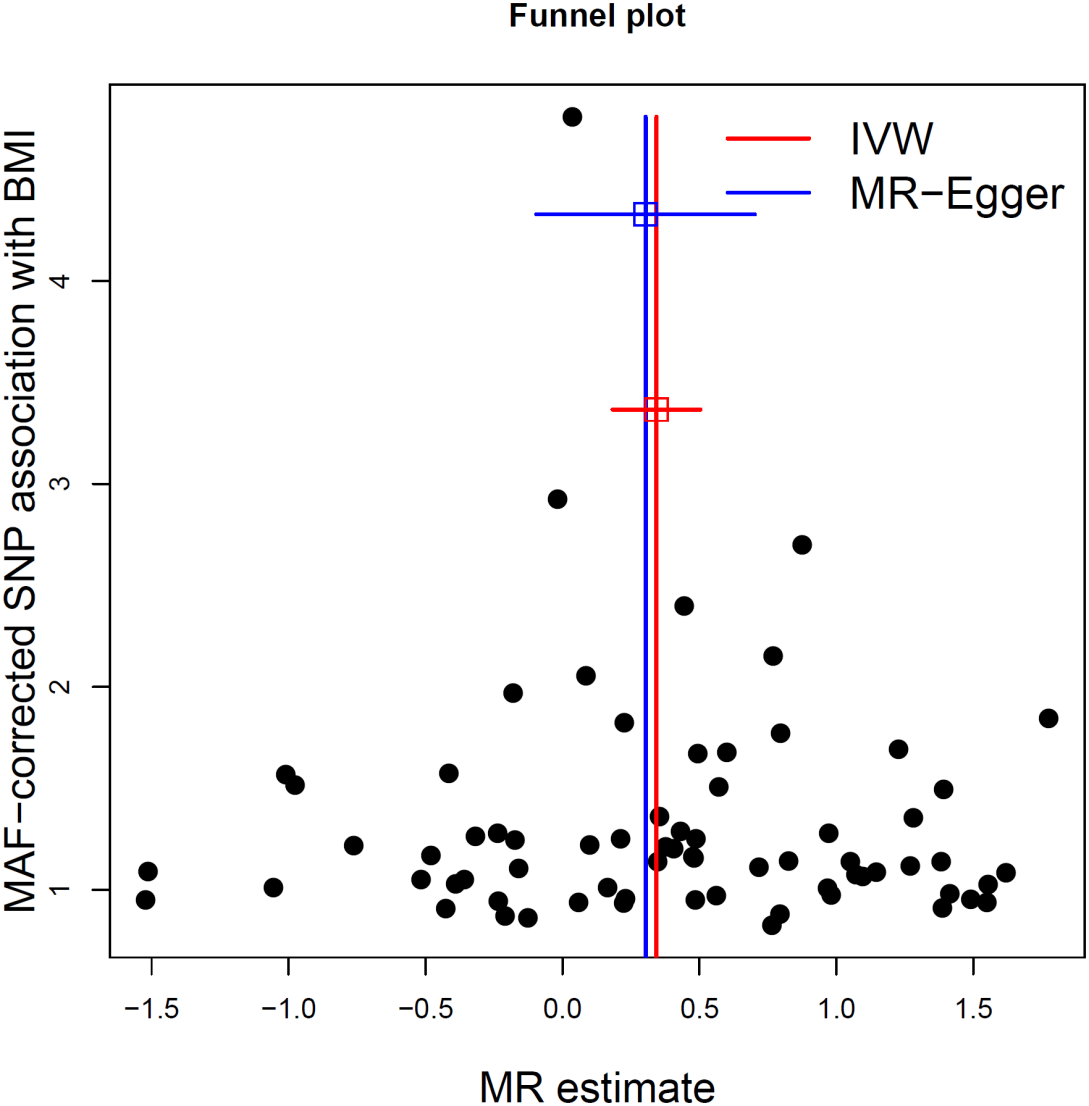
MR-Egger regression funnel plot for BMI on MS analysis. Each SNP’s MR estimate is plotted against its minor-allele frequency corrected association with BMI. A minor allele frequency (MAF) correction proportional to the SNP-BMI standard error is used since a low-frequency allele is likely to be measured with low precision. Similar to the use of funnel plots in the meta-analysis literature, this plot can be used for visual inspection of symmetry, where any deviation can be suggestive of pleiotropy. We note that our plot appears generally symmetrical.

We next tested whether any of the selected SNPs were influenced by LD, pleiotropy, or population stratification. First, none of the SNPs were found to be in LD with each other at an *r* ^2^ > 0.05. Second, the intercept term estimated from MR-Egger regression was centered at the origin with a confidence interval including the null (0.0013, 95% CI −0.010–0.013, *p* = 0.83) (Table 2), suggesting that pleiotropy had not unduly influenced the results. This observation was further confirmed by Fig 2, which represents a scatterplot of the effect estimates of SNPs associated with BMI and their corresponding effect on MS risk. The plot also displays the regression lines of our primary MR analysis (in red) and the MR-Egger adjusted for possible pleiotropy (in blue). Additionally, we inspected the funnel plot for any asymmetry, which would be suggestive of pleiotropy, and found the plot to the symmetrical (Fig 3). There is a higher prevalence of obesity among Southern and Eastern European populations [37,38]. Conversely, these populations have a decreased burden of MS relative to their Northern European counterparts [39]. Given the inversely correlated distributions of obesity and MS in Europe, the inclusion of SNPs potentially associated with ancestry within Europe would tend to bias our results towards the null (S2 Fig). Thus, residual effects of within-Europe ancestry would, on average, tend to underestimate true effects.

**Table 2:** Results of MR analyses and sensitivity analyses.

			MR Estimates			MR-Egger Regression	Weighted Median
Analysis	Number of SNPs	Number of Proxies	OR (95% CI)^a^	*p*-value	*I* ^2^ (95% CI)	Intercept (95% CI)	OR (95% CI)^a^
**All SNPs**	70	36	1.41 (1.20–1.66)	2.7 x 10^−5^	0% (0–29)	0.0013 (−0.010–0.013)	1.26 (0.98–1.62)
**Proxies with Allele Frequency 40% - 60%Excluded**	63	29	1.48 (1.25–1.74)	3.8 x 10^−6^	0% (0–30)	0.0046 (−0.0076–0.017)	1.42 (1.09–1.85)
**All Proxies Excluded**	34	0	1.65 (1.32–2.06)	1.2 x 10^−5^	0% (0–39)	0.012 (−0.0028–0.028)	1.52 (1.09–2.11)

^a^ OR, 95% CI for 1 SD increase in BMI.

### MR Estimates

**Fig 4:**
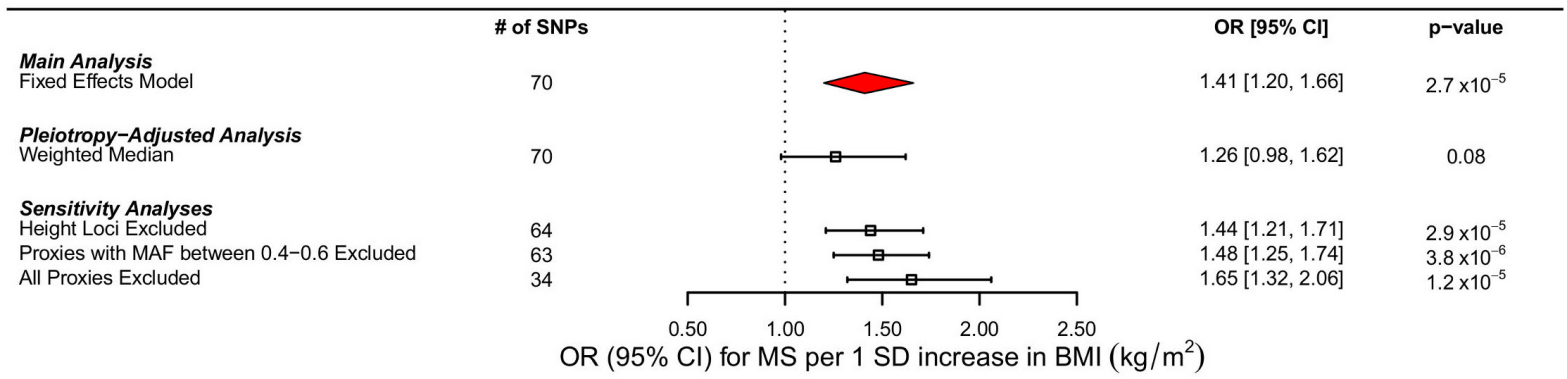
Forest plot of MR estimates. Forest plots of all main and sensitivity analyses. ORs for MS are reported for a 1 SD increase in BMI. Estimates were obtained using a fixed effects model. MAF refers to minor allele frequency.

Using MR analyses, a 1 standard deviation (SD) increase in BMI (kg/m^2^) was associated with a 41% increase in odds of MS (OR: 1.41, 95% CI 1.20–1.66, *p =* 2.72 x 10^−5^) (Table 2; Fig 4). The *I* ^2^ estimate for heterogeneity was 0% (95% CI 0%–29%). The slope of the MR-Egger regression was consistent with these findings (Fig 2). The results obtained using the weighted median approach further replicated this direction and magnitude of effect (OR: 1.26, 95% CI 0.98–1.62, *p* = 0.08), providing no evidence for pleiotropy.

In bidirectional MR analyses, we initially identified 110 SNPs reported by the IMSGC as genome-wide significant for MS; however, not all of these SNPs were ascertained directly in GIANT. Therefore, we used a total of 99 instruments for this analysis. Our results suggest that the genetic determinants of MS do not contribute to BMI (−0.0033 SD change in BMI per log odds increase in MS, 95% CI −0.011–0.0042, *p* = 0.39). The estimate for heterogeneity was large in this case (*I* ^*2*^: 43.5%, 95% CI 28.3%–55.6%). There was also no evidence of pleiotropy from the MR-Egger analyses (the intercept was centered at 0.002 with a 95% CI that included the origin [95% CI −0.0009–0.005, *p* = 0.17]) and an MR-Egger slope consistent with previous estimates (S3 and S4 Figs). Results from the weighted median analyses also were consistent with the main findings, decreasing the probability that pleiotropy influenced the results.

### Sensitivity Analyses

We excluded proxy SNPs to understand if random error, introduced by the use of imperfect proxies, had influenced our results. First, when all proxies were removed, we found a stronger relationship between BMI and MS, with a 1 SD change increasing odds of MS by 65% (OR: 1.65, 95% CI 1.32–2.06, *p* = 1.18 x10^-5^ (Table 2; Fig 4). Similarly, when we removed only proxies with allele frequencies between 0.4 and 0.6, we observed an OR of 1.48, similar to that obtained in the primary analysis (OR: 1.48, 95% CI 1.25–1.74, *p* = 3.82 x 10^−6^) (Table 2; Fig 4). In addition, we removed six SNPs that were in LD with known height loci (S1 Table), but again, this did not importantly change the results (OR: 1.44, 95% CI 1.21–1.71, *p* = 2.93 x 10^−5^) (Fig 4). MR results using only the IMSGC/WTCCC2 study were again concordant with our primary analysis (S1 Text and S2 Table).

## Discussion

Using an MR study design, these results provide evidence that genetically elevated BMI is strongly associated with an increased risk of MS, where a 1 SD increase in BMI conferred a 41% increase in the odds of MS. To place this in a clinical context, the mean SD for BMI reported among cohorts in the GIANT consortium was 4.70 kg/m^2^ [18]. Therefore, our estimates of a 41% increase in odds of MS correspond roughly to a change in BMI category from overweight (≥25 kg/m^2^) to obese (≥30 kg/m^2^) as per WHO obesity guidelines [40]. This suggests that childhood and early-adulthood BMI is an important modifiable risk factor for MS. The results of our bidirectional MR analysis indicate that MS does not influence BMI status.

The results of the MR analysis may offer some of the best evidence to assess the causal role of BMI in MS etiology since the results are less likely to be biased by confounding or reverse causation than traditional observational epidemiological study designs. By employing the two-sample MR approach, we were able to increase statistical power by selecting summary statistics from the largest GWAS studies for BMI (*n* = 322,105) and MS (up to *n* = 14,498 cases and 24,091 controls). Additionally, since genetic variants are stable over an individual’s life, our results represent lifetime risk of MS due to elevated BMI.

These findings may carry important public health implications because of the high prevalence of obesity in many countries [41]. For instance, results from the National Health and Nutrition Examination Survey (NHANES) suggest that approximately 17% of youth [42] and 35% of adults [43] living in the United States are considered to be obese. Therefore, the identification of elevated BMI as a susceptibility factor for MS places a high proportion of the population at a relatively higher risk for MS. Mean population BMI has increased in many Western countries over the past several decades [41], which coincides with rising MS incidence rates, particularly evident among females [44,45].

Elevated BMI has long been associated with an increased risk of many diseases, including those resulting in adverse cardiometabolic outcomes [46,47]; however, symptoms of these diseases typically do not manifest until the fifth or sixth decade of life [48,49]. In contrast, the median age of onset for MS is 28–31 y [50]; thus, our findings may demonstrate a more immediate consequence of elevated BMI. This provides further rationale to combat increasing youth obesity rates by implementing community and school-based interventions that promote physical activity and nutrition.

Current evidence suggests that there are several potential mechanisms through which increased BMI may affect MS risk; however, it remains unclear which of these pathways are critical. Vitamin D is a strong candidate, given that previous MR analyses demonstrate that genetically elevated BMI decreases 25-hydroxyvitamin D levels [51], and we have recently provided strong evidence supporting a causal role for reduced 25-hydroxyvitamin D levels as a risk factor for MS [30].

Elevated BMI has many physiological effects, and the exact mechanisms underlying the risk for MS conferred by an increased BMI remain unclear. For instance, obesity is known to promote a proinflammatory state [7–10], offering a potential mechanistic link to autoimmunity. In addition, results from a recent MR study show that increased BMI produces important effects on metabolite, lipoprotein, and hormone profiles [10]. In particular, adiponectin and leptin, the adipose-derived hormones, have been previously associated with MS and MS-related disability [52–54]. Under an obesogenic state, serum leptin levels rise whereas adiponectin levels fall, and this has been shown to reduce the production of regulatory T cells [55] and anti-inflammatory cytokines [56]. However, further research, such as prospective cohort studies or MR analyses, is required in order to understand the functional role of obesity in the risk of MS.

Our study also has limitations. First, while we undertook multiple sensitivity analyses to assess for pleiotropy, the possibility of residual pleiotropy is difficult to exclude. However, consistency across these approaches, and the fact that the MR-Egger intercept was centered at the origin, provides evidence that pleiotropy did not greatly influence the results. We can also not directly assess whether population stratification or compensatory mechanisms (otherwise known as canalization) may be influencing our results; however, these would likely bias our estimates towards the null [13]. While we ensured that our SNPs were not in LD with each other, it remains possible that they are in LD with SNPs that influence unknown risk factors for MS. Secondly, we note that both GIANT and the IMSGC used samples from the WTCCC cohort; therefore, we cannot exclude the possibility of sample overlap. This may have introduced bias into our results; however, given the sample size employed, this effect would likely be small since the WTCCC comprised ~2.5% of the overall GIANT consortium. Third, because of our use of summary-level statistics, we could not investigate nonlinear effects of BMI. Lastly, we cannot determine whether an established and more actionable intermediate, such as vitamin D, is primarily driving this causal relationship, nor can we conclude whether BMI influences MS progression.

In conclusion, these results provide evidence supporting a causal role for elevated BMI in MS etiology. This provides further rationale for individuals at risk for MS to maintain a healthy BMI. Whether vitamin D, or another established intermediate, is predominantly mediating this relationship warrants further investigation.

## Supporting Information

S1 Fig Flow diagram of SNP selection. This diagram shows the SNP selection process starting with the 77 SNPs that were genome-wide significant (*p* < 5 x10^-8^) for BMI in GIANT’s European sex-combined analysis. Thirty-four SNPs were genotyped directly in either the Immunochip or IMSGC/ WTCCC2 studies. An additional 36 proxies were identified with an r^2^ > 0.8. Therefore, we used a total of 70 SNPs for our MR analysis.
(JPG)

S2 Fig Schematic representation of residual European population stratification. This diagram is meant to illustrate how residual European population stratification may influence our results through the use of a directed acyclic graph. Since the literature reports that Northern European ancestry is associated with increased risk of MS but decreased BMI relative to Southern Europeans, the inclusion of BMI SNPs also associated with Northern European ancestry would tend to bias our estimates towards the null. This is similar to instances of negative confounding, where the presence of population stratification causes us to underestimate the true effect.
(TIF)

S3 Fig MR-Egger funnel plot for MS on BMI analysis. MR-Egger funnel plot for the bidirectional analysis of genetically increased MS risk on BMI.
(TIF)

S4 Fig MR-Egger scatterplot for MS on BMI analysis. The red line shows the standard MR estimate (IVW), and the blue line shows the pleiotropy-adjusted MR-Egger estimate for the change in SD BMI per increase in log odds of MS (−0.021, 95% CI −0.048–0.0058, *p* = 0.12). While it appears that the slopes of the two lines diverge, we note that the MR-Egger crosses the null and includes our original MR estimate within its 95% CI.
(TIF)

S1 Table BMI SNPs in LD with previously reported height loci. BMI SNPs that overlap with height loci previously reported in GIANT’s 2014 GWAS. SNPs were considered to be in LD if r^2^ > 0.05.
(DOCX)

S2 Table Characteristics of SNPs from IMSGC/WTCCC2-only sensitivity analysis. This table provides the genetic effect sizes and *p*-values for the association of the BMI SNPs with MS in the IMSGC/WTCCC2 study.
(DOCX)

S1 Text Results of IMSGC/WTCCC2-only sensitivity analysis. Additional text describing the results of the IMSGC/WTCCC2-only sensitivity analysis.
(DOCX)

## Abbreviations

BMI: body mass index
GIANT: Genetic Investigation of Anthropometric Traits
GWAS: genome-wide association studies
IMSGC: International Multiple Sclerosis Genetics Consortium
IVW: inverse-variance weighting
LD: linkage disequilibrium
MAF: minor allele frequency
MR: Mendelian randomization
MS: multiple sclerosis
NHANES: National Health and Nutrition Examination Survey
OR: odds ratio
SNP: single nucleotide polymorphism
WTCCC2: Wellcome Trust Case Control Consortium 2
