# Supplementary figures and images for: Obesity and Multiple Sclerosis: A Mendelian Randomization Study
Source: PLoS Med. 2016 Jun 28;13(6):e1002053. doi: 10.1371/journal.pmed.1002053 (PMC4924848; doi:10.1371/journal.pmed.1002053)

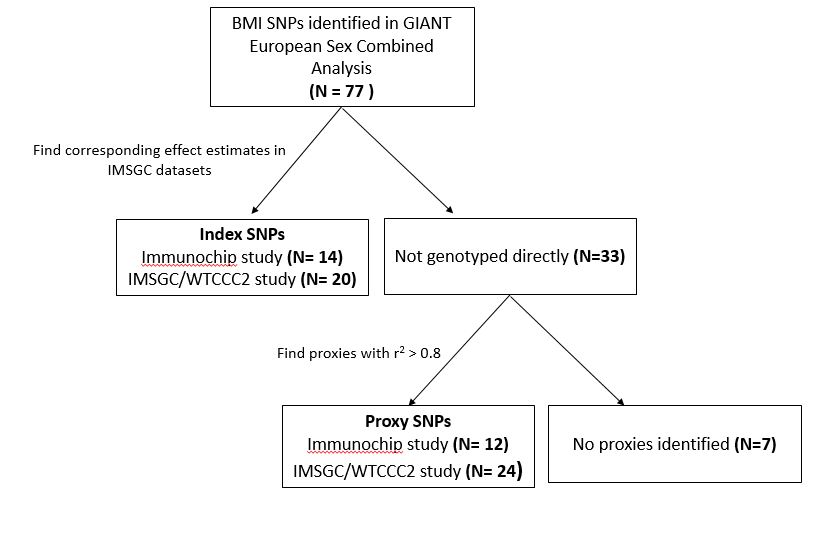

Supplement: S1 Fig — This diagram shows the SNP selection process starting with the 77 SNPs that were genome-wide significant (p < 5 x10^-8^) for BMI in GIANT’s European sex-combined analysis. Thirty-four SNPs were genotyped directly in either the Immunochip or IMSGC/ WTCCC2 studies. An additional 36 proxies were identified with an r2 > 0.8. Therefore, we used a total of 70 SNPs for our MR analysis. (JPG) [file pmed.1002053.s001.JPG]

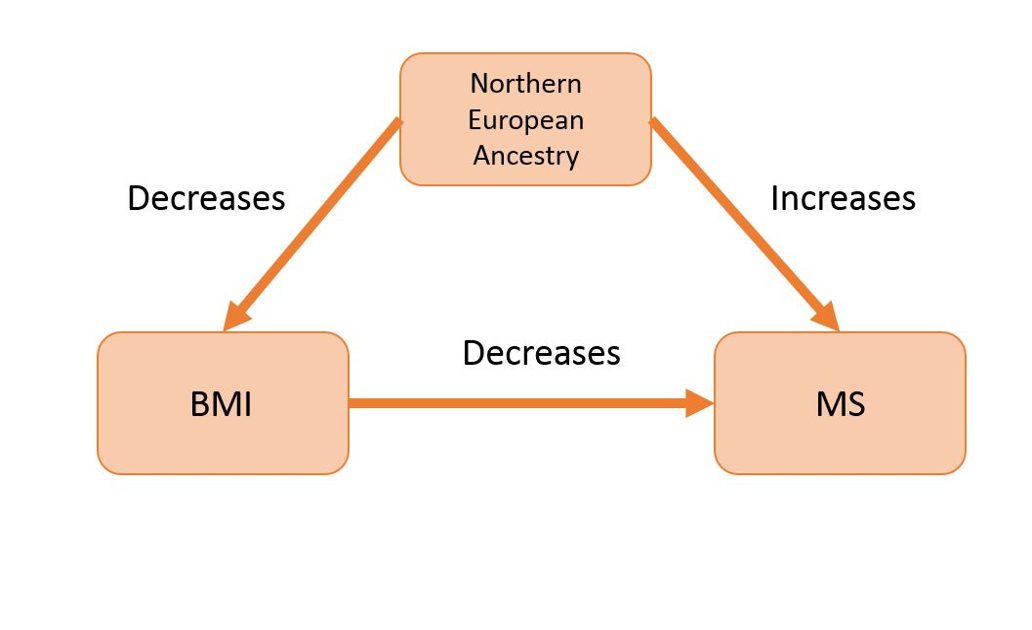

Supplement: S2 Fig — This diagram is meant to illustrate how residual European population stratification may influence our results through the use of a directed acyclic graph. Since the literature reports that Northern European ancestry is associated with increased risk of MS but decreased BMI relative to Southern Europeans, the inclusion of BMI SNPs also associated with Northern European ancestry would tend to bias our estimates towards the null. This is similar to instances of negative confounding, where the presence of population stratification causes us to underestimate the true effect. (TIF) [file pmed.1002053.s002.tif]

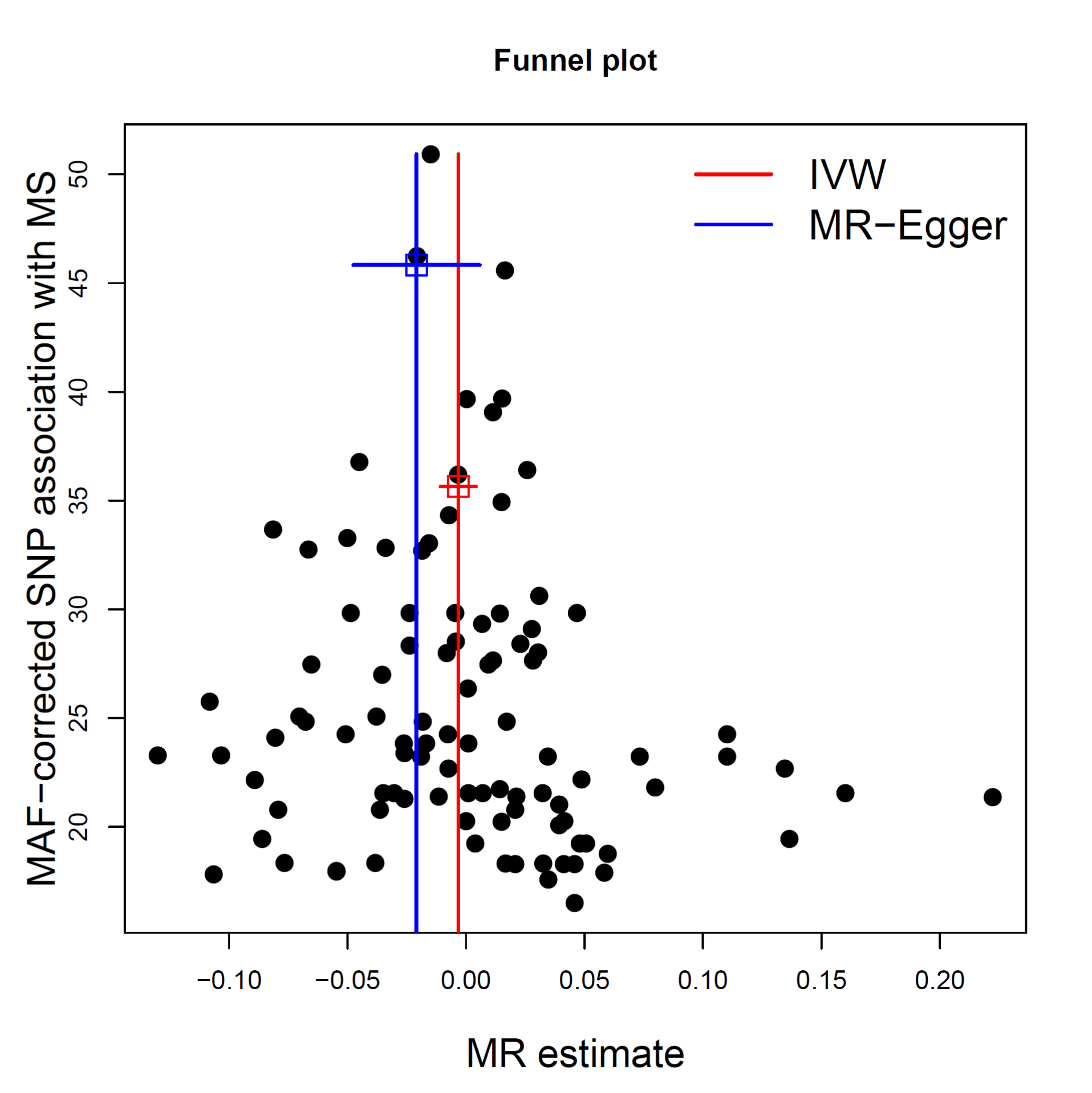

Supplement: S3 Fig — MR-Egger funnel plot for the bidirectional analysis of genetically increased MS risk on BMI. (TIF) [file pmed.1002053.s003.tif]

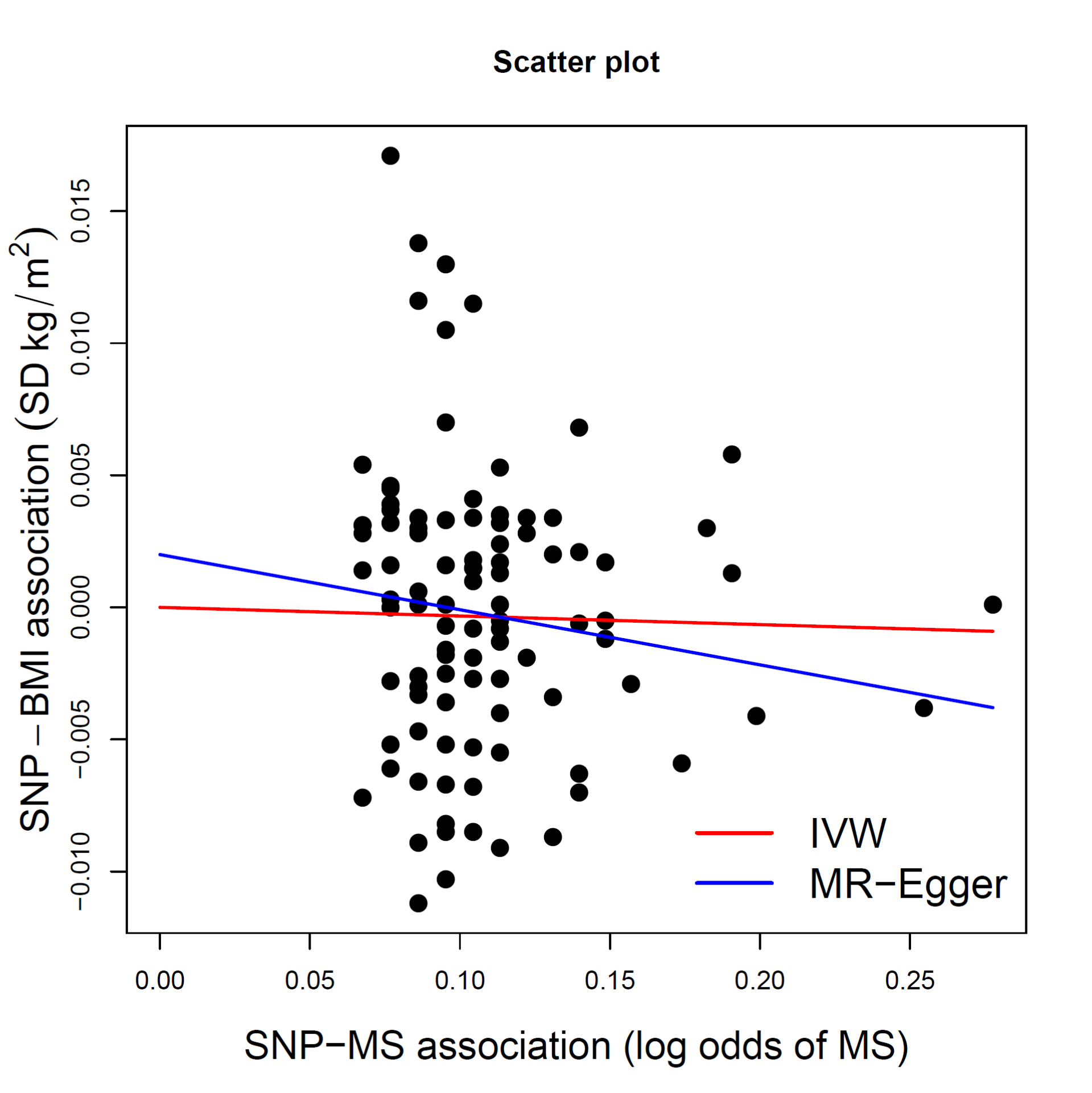

Supplement: S4 Fig — The red line shows the standard MR estimate (IVW), and the blue line shows the pleiotropy-adjusted MR-Egger estimate for the change in SD BMI per increase in log odds of MS (−0.021, 95% CI −0.048–0.0058, p = 0.12). While it appears that the slopes of the two lines diverge, we note that the MR-Egger crosses the null and includes our original MR estimate within its 95% CI. (TIF) [file pmed.1002053.s004.tif]
