# Supplementary material for: Obesity and Multiple Sclerosis: A Mendelian Randomization Study
Source: PLoS Med. 2016 Jun 28;13(6):e1002053. doi: 10.1371/journal.pmed.1002053 (PMC4924848; doi:10.1371/journal.pmed.1002053)
Supplement: S1 Table — BMI SNPs that overlap with height loci previously reported in GIANT’s 2014 GWAS. SNPs were considered to be in LD if r2 > 0.05. (DOCX) [file pmed.1002053.s005.docx]

**S1 Table: BMI SNPs in Linkage Disequilibrium with Previously Reported Height Loci**

| **BMI SNP** | **Height SNP** | **Distance (bp)** | **r^2^** |
| --- | --- | --- | --- |
| rs10182181 | rs2289195 | 313187 | 0.19 |
| rs12401738 | rs17391694 | 176865 | 0.18 |
| rs205262 | rs3800461 | 53158 | 0.29 |
| rs3817334 | rs1681630 | 318159 | 0.12 |
| rs6567160 | rs11152213 | 23813 | 0.96 |
| rs9400239 | rs479744 | 42369 | 0.68 |
