# Supplementary material for: Obesity and Multiple Sclerosis: A Mendelian Randomization Study
Source: PLoS Med. 2016 Jun 28;13(6):e1002053. doi: 10.1371/journal.pmed.1002053 (PMC4924848; doi:10.1371/journal.pmed.1002053)
Supplement: S2 Table — This table provides the genetic effect sizes and p-values for the association of the BMI SNPs with MS in the IMSGC/WTCCC2 study. (DOCX) [file pmed.1002053.s006.docx]

**S2 Table: Characteristics of SNPs from IMSGC/WTCCC2 Only Sensitivity Analysis**

|  | | | **Result from IMSCG/WTCCC2 Study**[20] | | | | | |
| --- | --- | --- | --- | --- | --- | --- | --- | --- |
| **BMI-associated SNP** | **Nearest Gene(s)** | ***p*-value^a^** | **OR** | **Lower**  **95% CI** | **Upper**  **95% CI** | ***p*-value** | **Proxy SNP** | ***r*^2 b^** |
| rs1558902 | *FTO* | 7.5x10^-153^ | 1.03 | 0.99 | 1.07 | 0.12 | rs9930333 | 0.91 |
| rs6567160 | *MC4R* | 3.9x10^-53^ | 1.01 | 0.96 | 1.05 | 0.75 | rs571312 | 0.99 |
| rs13021737 | *TMEM18* | 1.1x10^-50^ | 1.01 | 0.97 | 1.06 | 0.58 | rs4854344 | 0.99 |
| rs10938397 | *GNPDA2* | 3.2x10^-38^ | 1.00 | 0.97 | 1.04 | 0.80 | rs12641981 | 0.99 |
| rs543874 | *SEC16B* | 2.6x10^-35^ | 1.00 | 0.96 | 1.05 | 0.86 | rs633715 | 0.95 |
| rs2207139 | *TFAP2B* | 4.1x10^-29^ | 0.99 | 0.94 | 1.04 | 0.60 | rs4715210 | 1.00 |
| rs11030104 | *BDNF* | 5.6x10^-28^ | 1.03 | 0.99 | 1.08 | 0.16 | NA | NA |
| rs3101336 | *NEGR1* | 2.7x10^-26^ | 1.02 | 0.99 | 1.06 | 0.24 | rs7531118 | 0.80 |
| rs7138803 | *BCDIN3D* | 8.2x10^-24^ | 1.02 | 0.98 | 1.06 | 0.31 | NA | NA |
| rs10182181 | *ADCY3* | 8.8x10^-24^ | 1.04 | 1.00 | 1.08 | 0.07 | NA | NA |
| rs3888190 | *ATP2A1* | 3.1x10^-23^ | 1.01 | 0.98 | 1.05 | 0.48 | rs4788102 | 1.00 |
| rs1516725 | *ETV5* | 1.9x10^-22^ | 1.02 | 0.97 | 1.08 | 0.41 | rs6809651 | 0.99 |
| rs12446632 | *GPRC5B* | 1.5x10^-18^ | 1.06 | 1.00 | 1.12 | 0.04 | NA | NA |
| rs2287019 | *QPCTL* | 4.6x10^-18^ | 1.05 | 1.00 | 1.09 | 0.04 | NA | NA |
| rs16951275 | *MAP2K5* | 1.9x10^-17^ | 0.99 | 0.95 | 1.04 | 0.74 | rs2241423 | 0.99 |
| rs3817334 | *MTCH2* | 5.2x10^-17^ | 0.97 | 0.94 | 1.01 | 0.14 | rs7124681 | 0.99 |
| rs2112347 | *POC5* | 6.2x10^-17^ | 0.99 | 0.95 | 1.03 | 0.69 | rs34358 | 0.85 |
| rs12566985 | *FPGT-TNNI3K* | 3.3x10^-15^ | 0.99 | 0.96 | 1.03 | 0.76 | rs6604872 | 1.00 |
| rs3810291 | *ZC3H4* | 4.8x10^-15^ | 0.94 | 0.90 | 0.97 | 3.8 x 10^-4^ | rs10408163 | 1.00 |
| rs7141420 | *NRXN3* | 1.2x10^-14^ | 1.02 | 0.99 | 1.06 | 0.21 | NA | NA |
| rs13078960 | *CADM2* | 1.7x10^-14^ | 0.99 | 0.95 | 1.04 | 0.69 | rs7622475 | 0.99 |
| rs10968576 | *LINGO2* | 6.6x10^-14^ | 1.01 | 0.97 | 1.05 | 0.54 | NA | NA |
| rs12429545 | *OLFM4* | 1.1x10^-12^ | 1.01 | 0.96 | 1.06 | 0.79 | NA | NA |
| rs12286929 | *CADM1* | 1.3x10^-12^ | 0.99 | 0.95 | 1.03 | 0.57 | rs12421648 | 0.84 |
| rs11165643 | *PTBP2* | 2.1x10^-12^ | 1.01 | 0.97 | 1.05 | 0.69 | NA | NA |
| rs7903146 | *TCF7L2* | 1.1x10^-11^ | 0.98 | 0.95 | 1.02 | 0.35 | NA | NA |
| rs10132280 | *STXBP6* | 1.1x10^-11^ | 1.03 | 0.99 | 1.07 | 0.16 | rs8015400 | 0.90 |
| rs17405819 | *HNF4G* | 2.1x10^-11^ | 0.99 | 0.95 | 1.03 | 0.71 | rs2977345 | 0.97 |
| rs1016287 | *LINC01122* | 2.3x10^-11^ | 1.03 | 0.99 | 1.07 | 0.11 | rs759250 | 1.00 |
| rs4256980 | *TRIM66* | 2.9x10^-11^ | 0.99 | 0.95 | 1.03 | 0.69 | rs2316901 | 0.99 |
| rs17094222 | *HIF1AN* | 5.9x10^-11^ | 1.01 | 0.97 | 1.06 | 0.58 | rs17113301 | 0.90 |
| rs7599312 | *ERBB4* | 1.2x10^-10^ | 1.02 | 0.98 | 1.07 | 0.24 | NA | NA |
| rs2365389 | *FHIT* | 1.6x10^-10^ | 0.98 | 0.94 | 1.02 | 0.29 | rs7629340 | 0.98 |
| rs205262 | *C6orf106* | 1.8x10^-10^ | 0.99 | 0.95 | 1.03 | 0.54 | NA | NA |
| rs2820292 | *NAV1* | 1.8x10^-10^ | 1.03 | 0.99 | 1.07 | 0.11 | rs1032524 | 0.93 |
| rs12885454 | *PRKD1* | 1.9x10^-10^ | 1.01 | 0.98 | 1.05 | 0.42 | rs1307813 | 1.00 |
| rs12016871 | *MTIF3* | 2.3x10^-10^ | 1.01 | 0.97 | 1.06 | 0.58 | rs1885989 | 0.82 |
| rs16851483 | *RASA2* | 3.6x10^-10^ | 1.02 | 0.95 | 1.09 | 0.63 | rs2640017 | 0.99 |
| rs1167827 | *HIP1* | 6.3x10^-10^ | 0.96 | 0.93 | 1.00 | 0.04 | NA | NA |
| rs758747 | *NLRC3* | 7.5x10^-10^ | 1.03 | 0.99 | 1.07 | 0.16 | NA | NA |
| rs1928295 | *TLR4* | 7.9x10^-10^ | 1.02 | 0.99 | 1.06 | 0.25 | NA | NA |
| rs9925964 | *KAT8* | 8.1x10^-10^ | 0.96 | 0.92 | 1.00 | 0.03 | rs889548 | 1.00 |
| rs11126666 | *KCNK3* | 1.3x10^-9^ | 0.99 | 0.95 | 1.03 | 0.70 | NA | NA |
| rs2650492 | *SBK1* | 1.9x10^-9^ | 1.03 | 0.99 | 1.07 | 0.13 | NA | NA |
| rs6804842 | *RARB* | 2.5x10^-9^ | 1.03 | 0.99 | 1.07 | 0.15 | NA | NA |
| rs12940622 | *RPTOR* | 2.5x10^-9^ | 1.01 | 0.97 | 1.05 | 0.58 | NA | NA |
| rs4740619 | *C9orf93* | 4.6x10^-9^ | 1.00 | 0.96 | 1.04 | 0.86 | NA | NA |
| rs13191362 | *PARK2* | 7.3x10^-9^ | 1.01 | 0.95 | 1.07 | 0.83 | rs13202339 | 0.98 |
| rs3736485 | *DMXL2* | 7.4x10^-9^ | 0.98 | 0.94 | 1.01 | 0.22 | rs4775961 | 0.88 |
| rs17001654 | *SCARB2* | 7.7x10^-9^ | 1.02 | 0.97 | 1.07 | 0.47 | rs17001561 | 0.95 |
| rs11191560 | *NT5C2* | 8.5x10^-9^ | 0.97 | 0.91 | 1.03 | 0.29 | rs12411886 | 1.00 |
| rs1528435 | *UBE2E3* | 1.2x10^-8^ | 1.00 | 0.97 | 1.04 | 0.83 | rs6727573 | 0.95 |
| rs2075650 | *TOMM40* | 1.3x10^-8^ | 1.06 | 1.01 | 1.11 | 0.02 | NA | NA |
| rs1000940 | *RABEP1* | 1.3x10^-8^ | 1.01 | 0.97 | 1.05 | 0.64 | NA | NA |
| rs11583200 | *ELAVL4* | 1.5x10^-8^ | 1.01 | 0.98 | 1.05 | 0.48 | NA | NA |
| rs9400239 | *FOXO3* | 1.6x10^-8^ | 0.97 | 0.93 | 1.01 | 0.15 | rs2153960 | 0.93 |
| rs10733682 | *LMX1B* | 1.8x10^-8^ | 1.00 | 0.97 | 1.04 | 0.96 | NA | NA |
| rs11688816 | *EHBP1* | 1.9x10^-8^ | 1.02 | 0.99 | 1.06 | 0.21 | rs360791 | 0.82 |
| rs11057405 | *CLIP1* | 2.0x10^-8^ | 1.08 | 1.02 | 1.15 | 0.01 | NA | NA |
| rs2121279 | *LRP1B* | 2.3x10^-8^ | 1.02 | 0.96 | 1.08 | 0.53 | rs6714473 | 0.80 |
| rs29941 | *KCTD15* | 2.4x10^-8^ | 0.99 | 0.95 | 1.02 | 0.45 | NA | NA |
| rs3849570 | *GBE1* | 2.6x10^-8^ | 1.01 | 0.97 | 1.05 | 0.80 | rs7620240 | 1.00 |
| rs6477694 | *EPB41L4B* | 2.7x10^-8^ | 1.03 | 0.99 | 1.07 | 0.10 | NA | NA |
| rs2176598 | *HSD17B12* | 3.0x10^-8^ | 1.06 | 0.98 | 1.15 | 0.15 | rs11201714 | 1.00 |
| rs7899106 | *GRID1* | 3.0x10^-8^ | 1.03 | 0.99 | 1.07 | 0.16 | rs7110437 | 0.81 |
| rs17724992 | *PGPEP1* | 3.4x10^-8^ | 1.02 | 0.98 | 1.06 | 0.34 | NA | NA |
| rs7243357 | *GRP* | 3.9x10^-8^ | 0.99 | 0.95 | 1.04 | 0.70 | rs9961404 | 0.90 |
| rs1808579 | *C18orf8* | 4.2x10^-8^ | 1.00 | 0.96 | 1.04 | 0.91 | NA | NA |
| rs2033732 | *RALYL* | 4.9x10^-8^ | 1.00 | 0.96 | 1.04 | 0.83 | rs733594 | 0.91 |

^a^ p-value for association was obtained from GIANT consortium European sex-combined analysis[18]

^b^ r^2^ were estimated using UK10K European samples[21]
