# Supplementary material for: Obesity and Multiple Sclerosis: A Mendelian Randomization Study
Source: PLoS Med. 2016 Jun 28;13(6):e1002053. doi: 10.1371/journal.pmed.1002053 (PMC4924848; doi:10.1371/journal.pmed.1002053)
Supplement: S1 Text — Additional text describing the results of the IMSGC/WTCCC2-only sensitivity analysis. (DOCX) [file pmed.1002053.s007.docx]

**S1 Text: Results of IMSGC/WTCCC2 Only Sensitivity Analysis**

Corresponding effect estimates for 69 of the BMI SNPs were ascertained in the IMSGC/ WTCCC2 study (29 index SNPs and 40 proxies). MR estimates were produced as described in the Methods. This sensitivity analysis found that a one SD increase in genetically determined BMI increased odds of MS by 32% **(OR= 1.32, 95% CI= 1.11, 1.57, *p*= 1.37x10^-3^)**. The *I^2^* estimate for heterogeneity increased to 22% **(*I^2^* = 24%, 95% CI= 0% - 43%)**. These results are again concordant with our primary analysis. We note that the mean standard error of MS genetic effect sizes increased in this analysis since the IMSGC/WTCCC2 study included fewer samples. This may in part explain the increase in p-value.
